# Supplementary material for: Up-regulation of ubiquitin–proteasome activity upon loss of NatA-dependent N-terminal acetylation
Source: Life Sci Alliance. 2021 Nov 11;5(2):e202000730. doi: 10.26508/lsa.202000730 (PMC8605321; doi:10.26508/lsa.202000730)
Supplement: Supplementary file 1 [file LSA-2020-00730_TableS1.docx]

**Table S1: Yeast strains**

| Strain | Background | Genotype | Reference |
| --- | --- | --- | --- |
| FY1679 | S288c | MATa/α ura3-52/ura3-52 leu2Δ1/LEU2 his3Δ200/HIS3 trp1Δ63/TRP1 GAL2/GAL2 | [71] |
| ESM356-1 | FY1679 | MATa ura3-52 leu2Δ1 his3Δ200 trp1Δ63 | Elmar Schiebel |
| YCT1084 | ESM356-1 | ubr1Δ::hphNT1 | [72] |
| YMaM632 | ESM356-1 | naa20Δ::hphNT1 | [12] |
| YBB4 | ESM356-1 | ufd4Δ::hphNT1 | [12] |
| YBB5 | ESM356-1 | naa10Δ::hphNT1 | [12] |
| YBB9 | ESM356-1 | ufd4Δ::natNT2 ubr1Δ::hphNT1 | [12] |
| YBB52 | ESM356-1 | UFD4-3HA-kanMX6 | This study |
| YBB53 | ESM356-1 | naa10Δ::hphNT1 UFD4-3HA-kanMX6 | This study |
| YEO2 | ESM356-1 | naa10Δ::kanMX6 ubr1Δ::hphNT1 | This study |
| YEO3 | ESM356-1 | naa10Δ::kanMX6 ufd4Δ::natNT2 ubr1Δ::hphNT1 | [12] |
| YIK35 | ESM356-1 | naa10Δ::kanMX6 ufd4Δ::hphNT1 | This study |
| YIK55 | ESM356-1 | ufd2Δ::klURA3 | This study |
| YIK56 | ESM356-1 | ufd2Δ::klURA3 ufd4Δ::natNT2 ubr1Δ::hphNT1 | This study |
| YIK241 | ESM356-1 | ubr2Δ::klUra3 ufd4Δ::natNT2 ubr1Δ::hphNT1 | This study |
| YIK242 | ESM356-1 | ubr2Δ::klUra3 ufd4Δ::natNT2 ubr1Δ::hphNT1 naa10Δ::kanMX6 | This study |
| YIK278 | ESM356-1 | tom1Δ::klUra3 | This study |
| YIK280 | ESM356-1 | tom1Δ::klURA3 ufd4Δ::natNT2 ubr1Δ::hphNT1 | This study |
| YIK281 | ESM356-1 | tom1Δ::klURA3 naa10Δ::kanMX6 ufd4Δ::natNT2 ubr1Δ::hphNT1 | This study |
| YIK292 | ESM356-1 | ufd2Δ::natNT2 tom1Δ::klURA3 | This study |
| YIK300 | ESM356-1 | tom1Δ::natNT2 ubr1Δ::hphNT1 | This study |
| YIK301 | ESM356-1 | tom1Δ::natNT2 ufd4Δ::hphNT1 | This study |
| YIK305 | ESM356-1 | TOM1-TAP-kanMX4 | This study |
| YIK309 | ESM356-1 | RPN4-TAP-kanMX4 | This study |
| YIK311 | ESM356-1 | naa10Δ::natNT2 RPN4-TAP-kanMX4 | This study |
| YIK330 | ESM356-1 | pep4Δ0 | This study |
| YIK343 | ESM356-1 | natNT2-pGPD-FLAG-UFD4 pep4Δ0 | This study |
| YIK344 | ESM356-1 | natNT2-pGPD-FLAG-TOM1 pep4Δ0 | This study |
| YIK345 | ESM356-1 | natNT2-pGPD-FLAG-UBR1 pep4Δ0 | This study |
| YIK346 | ESM356-1 | natNT2-pGPD-FLAG-UFD2 pep4Δ0 | This study |
| YIK358 | ESM356-1 | pPRE4-sfGFP-KanMX-pPRE4-PRE4 | This study |
| YIK359 | ESM356-1 | pPRE5-sfGFP-KanMX-pPRE5-PRE5 | This study |
| YIK360 | ESM356-1 | pPRE6-sfGFP-KanMX-pPRE6-PRE6 | This study |
| YIK361 | ESM356-1 | pRPT3-sfGFP-KanMX-pRPT3-RPT3 | This study |
| YIK362 | ESM356-1 | pRPT5-sfGFP-KanMX-pRPT5-RPT5 | This study |
| YIK363 | ESM356-1 | pPUP1-sfGFP-KanMX-pPUP1-PUP1 | This study |
| YIK364 | ESM356-1 | pTUB1-sfGFP-KanMX-pTUB1-TUB1 | This study |
| YIK366 | ESM356-1 | pRPB2-sfGFP-KanMX-pRPB2-RPB2 | This study |
| YIK367 | ESM356-1 | naa10Δ::natNT2 pPRE4-sfGFP-KanMX-pPRE4-PRE4 | This study |
| YIK368 | ESM356-1 | naa10Δ::natNT2 pPRE5-sfGFP-KanMX-pPRE5-PRE5 | This study |
| YIK369 | ESM356-1 | naa10Δ::natNT2 pPRE6-sfGFP-KanMX-pPRE6-PRE6 | This study |
| YIK370 | ESM356-1 | naa10Δ::natNT2 pRPT3-sfGFP-KanMX-pRPT3-RPT3 | This study |
| YIK371 | ESM356-1 | naa10Δ::natNT2 pRPT5-sfGFP-KanMX-pRPT5-RPT5 | This study |
| YIK372 | ESM356-1 | naa10Δ::natNT2 pPUP1-sfGFP-KanMX-pPUP1-PUP1 | This study |
| YIK373 | ESM356-1 | naa10Δ::natNT2 pTUB1-sfGFP-KanMX-pTUB1-TUB1 | This study |
| YIK375 | ESM356-1 | naa10Δ::natNT2 pRPB2-sfGFP-KanMX-pRPB2-RPB2 | This study |
| YIK385 | ESM356-1 | pPRE4-sfGFP-KanMX-pPRE4-PRE4 Rpn4^A2N^ | This study |
| YIK386 | ESM356-1 | pPRE5-sfGFP-KanMX-pPRE5-PRE5 Rpn4^A2N^ | This study |
| YIK387 | ESM356-1 | pPRE6-sfGFP-KanMX-pPRE6-PRE6 Rpn4^A2N^ | This study |
| YIK388 | ESM356-1 | pRPT3-sfGFP-KanMX-pRPT3-RPT3 Rpn4^A2N^ | This study |
| YIK389 | ESM356-1 | pRPT5-sfGFP-KanMX-pRPT5-RPT5 Rpn4^A2N^ | This study |
| YIK390 | ESM356-1 | pPUP1-sfGFP-KanMX-pPUP1-PUP1 Rpn4^A2N^ | This study |
| YIK391 | ESM356-1 | pTUB1-sfGFP-KanMX-pTUB1-TUB1 Rpn4^A2N^ | This study |
| YIK393 | ESM356-1 | pRPB2-sfGFP-KanMX-pRPB2-RPB2 Rpn4^A2N^ | This study |
| YIK398 | ESM356-1 | naa10Δ::natNT2 pPRE4-sfGFP-KanMX-pPRE4-PRE4 Rpn4^A2N^ | This study |
| YIK399 | ESM356-1 | naa10Δ::natNT2 pPRE5-sfGFP-KanMX-pPRE5-PRE5 Rpn4^A2N^ | This study |
| YIK400 | ESM356-1 | naa10Δ::natNT2 pPRE6-sfGFP-KanMX-pPRE6-PRE6 Rpn4^A2N^ | This study |
| YIK401 | ESM356-1 | naa10Δ::natNT2 pRPT3-sfGFP-KanMX-pRPT3-RPT3 Rpn4^A2N^ | This study |
| YIK402 | ESM356-1 | naa10Δ::natNT2 pRPT5-sfGFP-KanMX-pRPT5-RPT5 Rpn4^A2N^ | This study |
| YIK403 | ESM356-1 | naa10Δ::natNT2 pPUP1-sfGFP-KanMX-pPUP1-PUP1 Rpn4^A2N^ | This study |
| YIK404 | ESM356-1 | naa10Δ::natNT2 pTUB1-sfGFP-KanMX-pTUB1-TUB1 Rpn4^A2N^ | This study |
| YIK406 | ESM356-1 | naa10Δ::natNT2 pRPB2-sfGFP-KanMX-pRPB2-RPB2 Rpn4^A2N^ | This study |
| YIK414 | ESM356-1 | ubr1Δ::klTrp1 ufd4Δ::hphNT1 Rpn4^A2N^ | This study |
| YIK415 | ESM356-1 | ubr1Δ::klTrp1 ufd4Δ::hphNT1 naa10Δ::natNT2 Rpn4^A2N^ | This study |
| YIK423 | ESM356-1 | tom1Δ::kanMX6 ufd4Δ::hphNT1 ubr1Δ::klTrp1 Rpn4^A2N^ | This study |
| YIK424 | ESM356-1 | tom1Δ::kanMX6 naa10Δ::natNT2 ufd4Δ::hphNT1 ubr1Δ::klTrp1 Rpn4^A2N^ | This study |
| YIK427 | ESM356-1 | Rpn4Δ(211-229)-TAP-kanMX4 | This study |
| YIK428 | ESM356-1 | Rpn4^A2N^Δ(211-229)-TAP-kanMX4 | This study |
| YIK429 | ESM356-1 | naa10Δ::hphNT1 Rpn4Δ(211-229)-TAP-kanMX4 | This study |
| YIK430 | ESM356-1 | naa10Δ::hphNT1 Rpn4^A2N^Δ(211-229)-TAP-kanMX4 | This study |
| YIK431 | ESM356-1 | naa20Δ::natNT2 Rpn4Δ(211-229)-TAP-kanMX4 | This study |
| YIK432 | ESM356-1 | naa20Δ::natNT2 Rpn4^A2N^Δ(211-229)-TAP-kanMX4 | This study |
| YIK460 | ESM356-1 | ubr1Δ::kanMX6 ufd4Δ::natNT2 Rpn4Δ(211-229) | This study |
| YIK462 | ESM356-1 | ubr1Δ::kanMX6 ufd4Δ::natNT2 naa10Δ::hphNT1 Rpn4Δ(211-229) | This study |
| YIK464 | ESM356-1 | ubr2Δ::klUra3 ubr1Δ::kanMX6 ufd4Δ::natNT2 Rpn4Δ(211-229) | This study |
| YIK466 | ESM356-1 | ubr2Δ::klUra3 ubr1Δ::kanMX6 ufd4Δ::natNT2 naa10Δ::hphNT1 Rpn4Δ(211-229) | This study |
| YIK469 | ESM356-1 | pdr5Δ::kanMX6 ubr2Δ::natNT2 | This study |
| YIK470 | ESM356-1 | pdr5Δ::kanMX6 ubr2Δ::natNT2 naa10Δ::hphNT1 | This study |
| YIK471 | ESM356-1 | pdr5Δ::kanMX6 ubr2Δ::natNT2 naa20Δ::hphNT1 | This study |
| YIK476 | ESM356-1 | pIK117 in YIK469 | This study |
| YIK477 | ESM356-1 | pIK118 in YIK469 | This study |
| YIK478 | ESM356-1 | pIK117 in YIK470 | This study |
| YIK479 | ESM356-1 | pIK118 in YIK470 | This study |
| YIK480 | ESM356-1 | pIK117 in YIK471 | This study |
| YIK481 | ESM356-1 | pIK117 in YIK471 | This study |
| YIK585 | ESM356-1 | natNT2-pGPD-UFD4 | This study |
| YIK586 | ESM356-1 | natNT2-pGPD-TOM1 | This study |
| YIK587 | ESM356-1 | natNT2-pGPD-UBR1 | This study |
| YIK619 | ESM356-1 | naa10Δ::natNT2 TOM1-TAP-kanMX4 | This study |
| YIK644 | ESM356-1 | leu2Δ::pGPD-mCherry-tCYC1-hphNT1 TOM1-sfGFP-kanMX | This study |
| YIK645 | ESM356-1 | leu2Δ::pGPD-mCherry-tCYC1-hphNT1 naa10Δ::natNT2 TOM1-sfGFP-kanMX | This study |
| YCHR76 | ESM356-1 | natNT2-pGal1-NAA10 | This study |
| YCHR77 | ESM356-1 | natNT2-pGal1-NAA10 ufd4*∆*::hphNT1 | This study |
