# Supplementary material for: Up-regulation of ubiquitin–proteasome activity upon loss of NatA-dependent N-terminal acetylation
Source: Life Sci Alliance. 2021 Nov 11;5(2):e202000730. doi: 10.26508/lsa.202000730 (PMC8605321; doi:10.26508/lsa.202000730)
Supplement: Supplementary file 2 [file LSA-2020-00730_TableS2.docx]

**Table S2: Plasmids**

| Plasmid | Description | Reference |
| --- | --- | --- |
| pET28c | E. coli expression vector | Novagen |
| pFA6a-kanMX6 | template for gene deletion by PCR targeting with kanMX6 selection marker | ([Wach et al., 1994](#_ENREF_82)) |
| pFA6a-hphNT1 | template for gene deletion by PCR targeting with hphNT1 selection marker | ([Janke et al., 2004](#_ENREF_31)) |
| pFA6a-natNT2 | template for gene deletion by PCR targeting with natNT2 selection marker | ([Janke et al., 2004](#_ENREF_31)) |
| pYM13 | Template for C-terminal tagging with TAP-tag by PCR targeting with kanMX6 selection marker | ([Janke et al., 2004](#_ENREF_31)) |
| pYM23 | Template for C-terminal tagging with 3Myc-tag by PCR targeting with klTrp1 selection marker | ([Janke et al., 2004](#_ENREF_31)) |
| pRS413 | CEN ARS HIS3 | ([Sikorski and Hieter](#_ENREF_70)) |
| p413-GPD | *CEN ARS HIS3* pGPD-tCYC1 | ([Mumberg et al.](#_ENREF_55)) |
| pArd1 | pRS416-NAA10 | Ulrike Friedrich |
| pGR295 | p415-TEF-10xHis-Ubi | ([Khmelinskii et al., 2014](#_ENREF_39)) |
| pAnB19 | pRS413-pGPD-Ubi-EcoRV-STOP-e^K^-mCherry-sfGFP | ([Kats et al., 2018](#_ENREF_37)) |
| pAnB19-PP | pRS413-pGPD-Ubi-PP-e^K^-mCherry-sfGFP | ([Kats et al., 2018](#_ENREF_37)) |
| pAnB19-EH | pRS413-pGPD-Ubi-EH-e^K^-mCherry-sfGFP | ([Kats et al., 2018](#_ENREF_37)) |
| pAnB19-Ubi^G76V^ | pRS413-pGPD-Ubi^G76V^-e^K^-mCherry-sfGFP | ([Kats et al., 2018](#_ENREF_37)) |
| pIK35 | pFA6a-klUra3 | ([Kats et al., 2018](#_ENREF_37)) |
| pIK41 | pRS413-pGPD-Ubi^G76V^-e^K^-mCherry-sfGFPcp8 | This study |
| pIK45 | pRS413-pGPD-Ubi^K48R,G76V^-e^K^-mCherry-sfGFP | This study |
| pIK57 | pRS413-pGPD-Rpn4(1-80)-e^K^-mCherry-sfGFP | This study |
| pIK59 | template for pGPD-driven overexpression and N-terminal tagging with FLAG-tag by PCR targeting with natNT2 selection marker | This study |
| pIK66 | pRS413-pGPD-Rpn4^A2N^(1-80) -e^K^-mCherry-sfGFP | This study |
| pIK78 | 6xHis-Ubc4 in pET28c | This study |
| pIK79 | 6xHis-Rad6 in pET28c | This study |
| pIK100 | Ubi-ProtA-6xHis in pET28c | This study |
| pIK102 | Ubi^K48R^-ProtA-6xHis in pET28c | This study |
| pIK117 | p413-GPD-Rpn4^C477A^-10xHis-sfGFPcp8 | This study |
| pIK118 | p413-GPD-Rpn4^A2N,C477A^-10xHis-sfGFPcp8 | This study |
